# The association between internet use and the choice of medical institution among Chinese older adults

**DOI:** 10.1186/s12877-024-04994-3

**Published:** 2024-06-21

**Authors:** Liuying Wang, Zirong Cheng, Li Ye, Lijuan Rong, Ching-Wen Chien, Tao-Hsin Tung

**Affiliations:** 1 Institute for Hospital Management, Tsinghua University, Shenzhen Campus, Shenzhen, 518055 China; 2 Evidence-based Medicine Center, Taizhou Hospital of Zhejiang Province Affiliated to Wenzhou Medical University, Linhai, Zhejiang 317000 China; 3 Key Laboratory of evidence-based Radiology of Taizhou, Linhai, Zhejiang 317000 China; 4 Taizhou Institute of Medicine, Health and New Drug Clinical Research, Zhejiang, 31700 China

**Keywords:** Internet, Health care provider choice, Hierarchical medical policy, Older adults

## Abstract

**Background:**

As older people have complex medical needs and still encounter challenges in accessing online health information, the relationship between Internet use and the choice of medical institution made by them is unclear, and we aimed to examine this relationship.

**Methods:**

Data from the newly released 2020 China Family Panel Survey database were used. Furthermore, we used descriptive statistics to analyze the background characteristics of the sample and a logistic regression model to estimate the impact of Internet use on the choice of medical institution made by older adults. We conducted a stratified analysis to explore the influence of different characteristics on the relationship between Internet use and the choice of medical institution.

**Results:**

Totally 4,948 older adults were included. Multivariate logistic regression showed that, compared to non-Internet users, Internet users were less likely to choose community health service centers over general hospitals (*P* < 0.001, OR = 0.667, 95CI%: 0.558–0.797). The subgroup analyses found that Internet use only had an impact on the choice of medical institution in older adults aged 65–69 years, those with partners, those with primary or secondary education, those residing in urban areas, those without medical insurance, those with a self-rated health status as average or healthy, those with unchanged or better health trend, and those without chronic disease. The effect of Internet use on the choice of medical institution did not differ by sex, satisfaction, or trust in doctors.

**Conclusion:**

Internet use may significantly affect older adults’ tendency to choose general hospitals to meet their daily medical needs. The subgroup analyses indicated that different characteristics of older people affected this association.

## Introduction

Older people comprise the primary group in health service utilization due to their decline in physical function and high prevalence of chronic diseases [1, 2]. At present, the rate of aging of the Chinese population is rising, the social medical burden is increasing, and China’s medical service system is facing great challenges and pressure. The UK, the US, Germany, Japan, and other countries have established relatively mature hierarchical diagnosis and treatment systems after years of attempts [3–6]. However, in China, owing to the imbalance of high-quality resources in general hospitals and the policy arrangement for non-compulsory primary diagnosis [7], there are difficulties in the implementation of primary diagnosis, leading to serious wastage of health resources [8, 9]. Many studies have explored the factors that affect residents’ choice of medical treatment. From the perspective of suppliers, care providers’ ability, mode of service, cost, and travel time affect patients’ choice of medical treatment [10]. From the perspective of demand, age, education level, and medical insurance also affect patients’ medical preferences [11, 12].

With the rapid popularization of the Internet in China, the Chinese government proposed a development strategy for *Internet & health care* in 2018 and regarded it as an important means to promote the implementation of a hierarchical medical system and optimize the allocation of medical resources [13]. Scholars have also begun to pay attention to the impact of Internet use on patients’ medical institution choices. Previous studies have focused on the relationship between online health information and the healthcare choices of adult patients. Internet information spillover could change the choice of hospital in patients undergoing benign surgeries in Korea [14]. However, the impact of the Internet is limited because patients have different preferences for accessing healthcare information [15]. Chinese scholars Liu et al. reported that the overflow of Internet medical information caused polarization in adults’ choice of self-diagnosis and high-grade hospitals [16]. Similarly, in a study by Ma et al., adults who participated in online browsing activities were 1.86 times more likely to choose municipal healthcare over primary care [17]. However, there is limited evidence on the influence of the Internet on hospital choices in older patients. A survey on people aged ≥ 45 years demonstrated that Internet use made patients suffering from common diseases show a more positive tendency to self-treat and choose top hospitals [18]. As older people have complex medical needs and they encounter challenges in accessing online health information [19–22], the relationship between Internet use and the choice of medical institution among them is unclear. Therefore, this study intends to use data from the 2020 China Family Panel Survey (CFPS) to explore the association between Internet use and the choice of medical institution among people aged ≥ 60 years and conduct subgroup analyses to explore the differences in the impacts of different characteristics.

## Materials and methods

### Data sources

The China Family Panel Studies (CFPS) is a nationally representative annual longitudinal survey of Chinese communities, families, and individuals launched in 2010 by the Institute of Social Science Survey of Peking University, China. It aims to track and collect data reflecting changes in Chinese society, economy, demographics, education, and health. Interviews were conducted using computer-assisted personal interviewing technology provided by the Survey Research Center (SRC) at the University of Michigan. The survey management system helps reduce measurement errors and monitors interview quality.

**Fig. 1:**
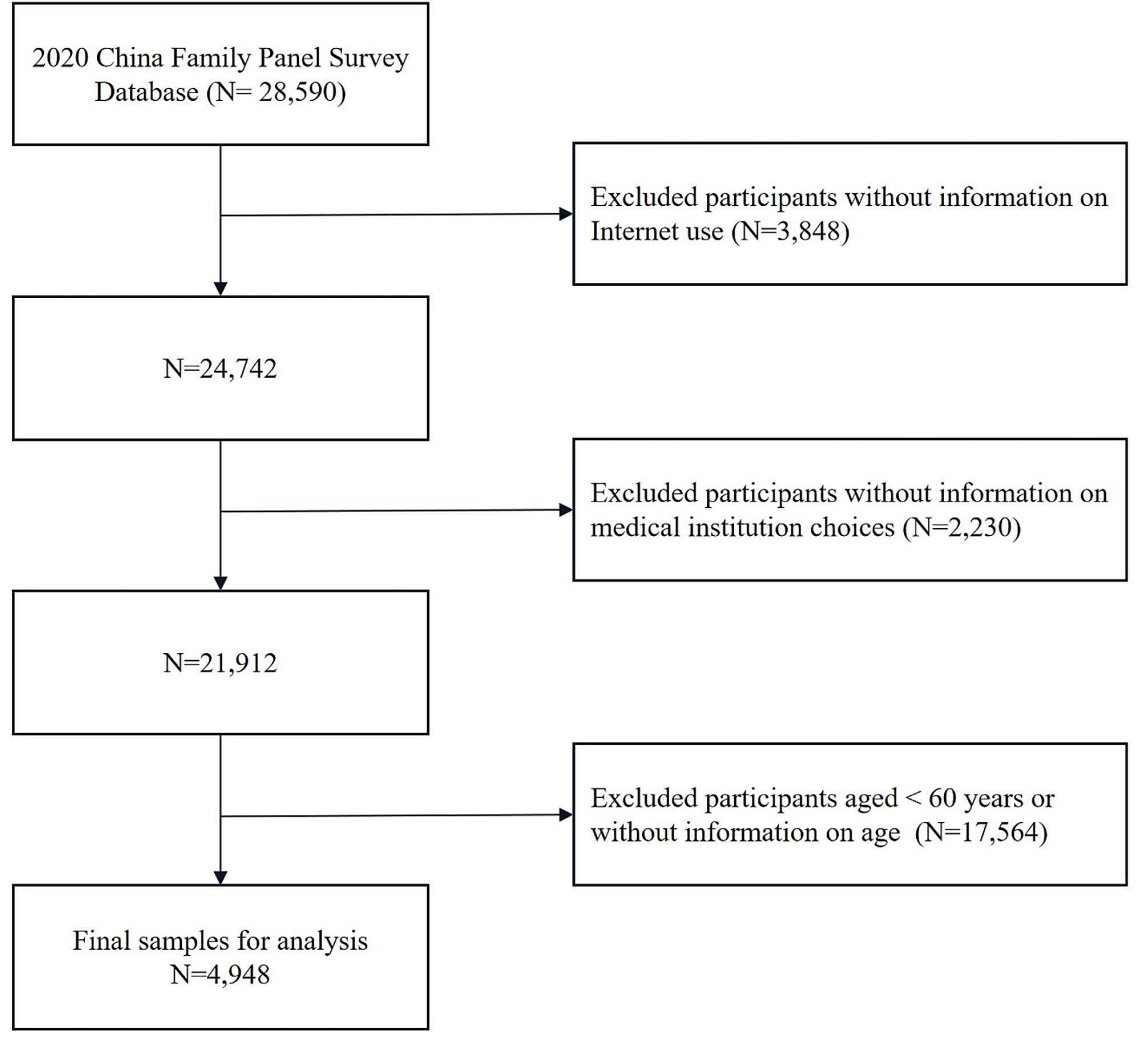
Flow diagram of participant recruitment

We conducted a cross-sectional study using the CFPS data collected in 2020. We excluded the following participants: those who did not provide information on Internet use and medical institution choices, those who were under 60 years old, and those who did not provide information on age. The final dataset included 4,948 older adults (Fig. 1).

This study was exempted from informed consent and was approved by the Ethics Committee of Taizhou Hospital of Zhejiang Province (approval number: K20230841). All procedures were performed in accordance with the guidelines of the institutional ethics committee and adhered to the tenets of the Declaration of Helsinki. All participant information was anonymously maintained.

### Dependent variable

The dependent variable was the choice of the medical institution. This was measured by enquiring about the kind of medical institution usually chosen by them when they sought medical treatment. There were five choices: general hospitals, specialized hospitals, community health service centers/township health centers, community health service stations/village clinics, and clinics. “Community health service centers/township health centers” and “community health service stations/village clinics” referred to medical facilities that treated common diseases. In this study, they were combined into “community health service centers.” “Clinics” and “specialized hospitals” were merged into “other medical institutions.” Medical institution choices were divided into three categories: general hospitals, community health service centers, and other medical institutions.

### Independent variable

As most older people in China are light users of the Internet, their proactive engagement with online content is limited, and they are mostly passively exposed to health information [12, 18, 19]. In this study, Internet use was indicated when the older person received health information through the Internet. This was determined by asking the respondents whether they used mobile devices or computers to access the Internet. Internet use was a binary variable. Individuals who did not use mobile devices or computers to access the Internet were classified as non-Internet users, whereas those who did were classified as Internet users.

Variables that could have impacted medical institution choices were considered. The demographic factors included age, sex, marital status, education, annual income, and residential area. Satisfaction with medical care and trust in doctors were analyzed as attitudes toward the medical services. The health-related characteristics included self-rated health status, health trend and chronic disease.

### Statistical analysis

**Fig. 2:**
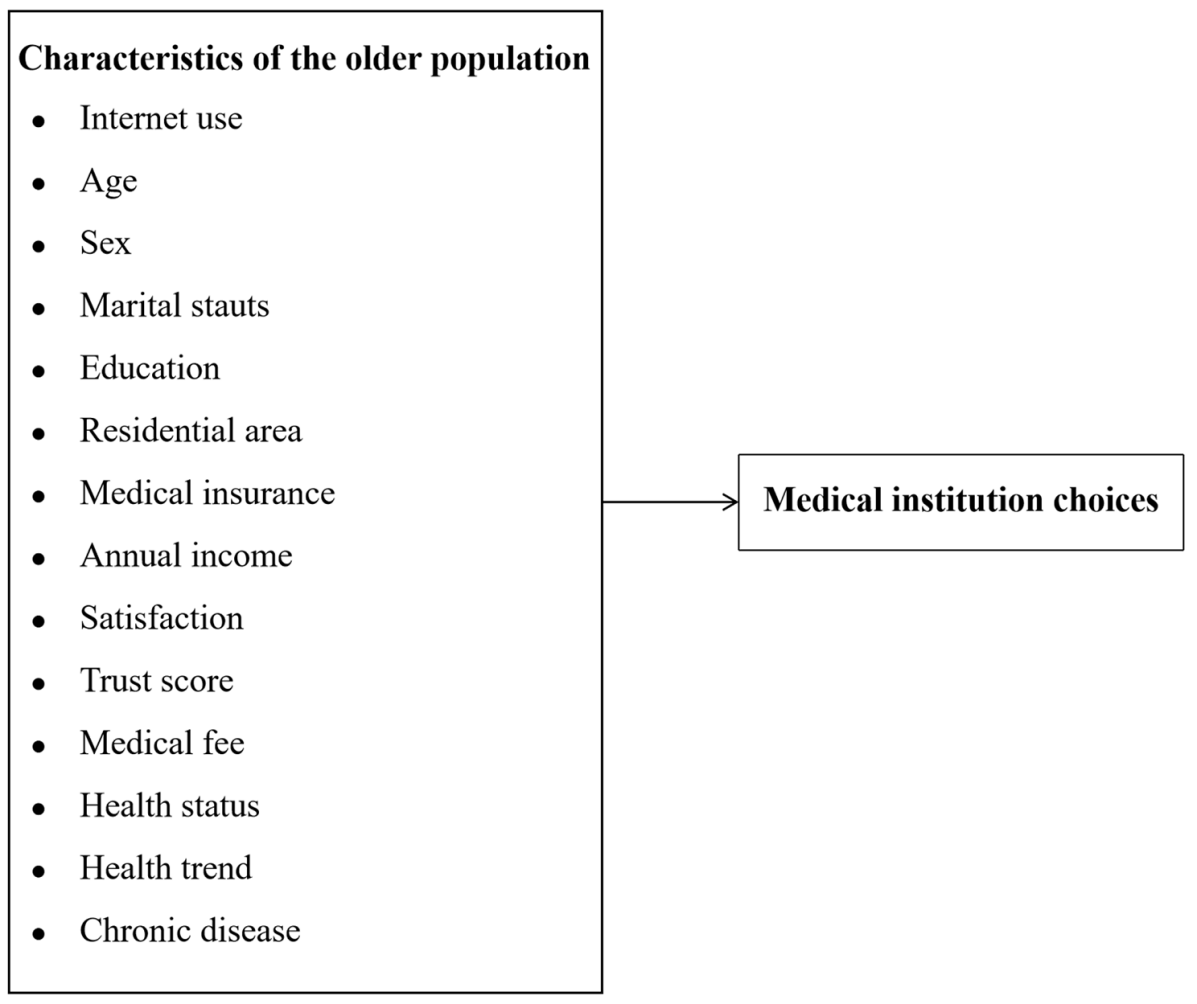
Research framework

Descriptive statistics were used to characterize the basic attributes of the study population. Chi-square test was used for categorical variables to determine associations, whereas variance analyses were used for continuous variables. A multivariate logistic regression analysis adjusted for confounding factors was used to evaluate the association between Internet use and medical institution choice. General hospitals were used as the reference group, because the outcome variables were divided into three categories. Subgroup analyses were performed to investigate the differences among older adults with different characteristics after adjusting for confounding factors. All analyses were performed using Stata MP 17. The significance threshold was set at *P* < 0.05 (Fig. 2).

## Results

### Population characteristics

The study sample comprised 4,948 participants. The prevalence of Internet use among people aged ≥ 60 years was 22.2%. Internet use was equally distributed among different age groups, with the highest being in the 65–69 age group (33.4%). More than 80% of the participants had completed middle high school or lower, 50.4% lived in rural areas, 49.6% lived in urban areas, and 91.6% had no medical insurance. The average annual income of the participants ranged from CNY 15,000–40,000. The participants were generally satisfied with their healthcare institutions and trusted their doctors. The health status of > 70% of the older people was mainly “general” and “healthy”. In addition, the health status of 90.9% of older adults had stabilized or improved in the past year, and 30.1% had chronic diseases. Table 1 presents the characteristics of the participants categorized by medical institution. Of the participants, 39.7% and 37.7% chose community health service centers and general hospitals, respectively. Compared to participants who chose community health service centers and other medical institutions, those who chose general hospitals were more likely to live in urban areas with higher annual income, poorer health status, and chronic diseases.

### Logistic analyses

Multivariate logistic regression results showed that, compared to non-Internet users, Internet users were less likely to choose community health service centers over general hospitals (*P* < 0.001, OR = 0.667, 95CI%: 0.558–0.797). However, the influence of Internet use on the choice of other medical institutions and general hospitals among the older people was not statistically significant (*P* = 0.118, OR = 0.851, 95CI%: 0.6955–1.042) (Table 2).

Other variables that decreased the tendency of choosing community health service centers included age 70–74 years (OR = 0.816, 95CI%: 0.673–0.989), age ≥ 75 years (OR = 0.633, 95CI%: 0.510–0.785), higher education level (primary & middle School vs. no education OR = 0.810, 95CI%: 0.694–0.945; high school and above vs. no education OR = 0.625, 95CI%: 0.497–0.787), higher annual income (OR = 0.977, 95CI%: 0.959–0.997), and higher medical fee (OR = 0.936, 95CI%: 0.918–0.954), whereas male sex (OR = 1.259, 95CI%: 1.093–1.451), no partner (OR = 1.241, 95CI%: 1.017–1.515), higher trust score (6–8 score vs. 0–5 score OR = 1.367, 95CI%: 1.160–1.610; 9–10 score vs. 0–5 score OR = 1.295, 95CI%: 1.085–1.545), and better health status (average health vs. unhealthy OR = 1.442, 95CI%: 1.210–1.719) were associated with a positive tendency to choose community health service centers (Table 2).

### Subgroup analysis

The association between Internet use and medical institution choice is examined in Table 3 using a subgroup analysis stratified by age, sex, marital status, education, residential area, medical insurance, *annual* income, satisfaction, trust score, medical fee, health status, health trend, and chronic disease. Internet use only had an impact on the choice of medical institution in older adults aged 65–69 (*P*<0.001, OR = 0.531, 95CI%: 0.387–0.729), those with partners (*P*<0.001, OR = 0.633, 95CI%: 0.523–0.766), those with primary or secondary education (*P*<0.001, OR = 0.605, 95CI%: 0.474–0.773), those residing in an urban area (*P*<0.001, OR = 0.576, 95CI%: 0.458–0.725), those without medical insurance (*P*<0.001, OR = 0.654, 95CI%: 0.545–0.786), those with self-rated health status as average (*P*<0.001, OR = 0.621, 95CI%: 0.491–0.786) or healthy (*P* = 0.008, OR = 0.591, 95CI%: 0.400–0.874), those with health trend reported as unchanged (*P*<0.001, OR = 0.620, 95CI%: 0.487–0.789) or better (*P* = 0.009, OR = 0.673, 95CI%: 0.499–0.907), and those with no chronic disease (*P*<0.001, OR = 0.646, 95CI%: 0.521–0.801). The effect of Internet use on the choices of medical institution did not differ statistically by sex, satisfaction or trust in the doctor for all stratified results.

**Table 1:** The characteristics of participants

Variable	Categories	General hospitals (*n* = 1867)	Community health service centers (*n* = 1965)	Other medical institutions (*n* = 1116)	Total	*P*
Internet use	No	1352(72.4)	1622(82.5)	874(78.3)	3848(77.8)	**<0.001**
	Yes	515(27.6)	343(17.5)	242(21.7)	1100(22.2)	
Age (year)	60–64	529(28.3)	609(31)	363(32.5)	1501(30.3)	**<0.001**
	65–69	598(32)	659(33.5)	393(35.2)	1650(33.4)	
	70–74	401(21.5)	414(21.1)	223(20)	1038(21.0)	
	≥ 75	339(18.2)	283(14.4)	137(12.3)	759(15.3)	
Sex	Female	914(49)	870(44.3)	608(54.5)	2392(48.3)	**<0.001**
	Male	953(51)	1095(55.7)	508(45.5)	2556(51.7)	
Marital status	Partnered	1567(83.9)	1634(83.2)	895(80.2)	4096(82.8)	0.063
	Non-partnered	253(13.6)	287(14.6)	183(16.4)	723(14.6)	
	Others	47(2.5)	44(2.2)	38(3.4)	129(2.6)	
Education	Uneducated	673(36)	858(43.7)	481(43.1)	2012(40.7)	**<0.001**
	Primary and middle school	837(44.8)	862(43.9)	460(41.2)	2159(43.6)	
	High school and above	357(19.1)	245(12.5)	175(15.7)	777(15.7)	
Residential area	Rural	744(39.9)	1142(58.1)	610(54.7)	2496(50.4)	**<0.001**
	Urban	1123(60.1)	823(41.9)	506(45.3)	2452(49.6)	
Medical insurance	No	1715(91.9)	1825(92.9)	993(89)	4533(91.6)	**0.001**
	Yes	152(8.1)	140(7.1)	123(11)	415(8.4)	
Annual income (CNY)	Continuous variable	20495.5(± 26916.9)	12527.2(± 22215.5)	12738.0(± 21207.7)	15581.4(± 24191.7)	**<0.001**
Satisfaction	Dissatisfied	141(7.6)	123(6.3)	73(6.5)	337(6.8)	0.11
	Neutral	223(11.9)	257(13.1)	166(14.9)	646(13.1)	
	Satisfied	1503(80.5)	1585(80.7)	877(78.6)	3965(80.1)	
Trust score	0–5 score	589(31.5)	504(25.6)	368(33)	1461(29.5)	**<0.001**
	6–8 score	741(39.7)	816(41.5)	390(34.9)	1947(39.4)	
	9–10 score	537(28.8)	645(32.8)	358(32.1)	1540(31.1)	
Medical fee (CNY)	Continuous variable	8994.6(± 24421.2)	2940.6(± 8155.5)	4297.7(± 13040.9)	5531.0(± 17240.4)	**<0.001**
Health status	Unhealthy	555(29.7)	421(21.4)	308(27.6)	1284(26.0)	**<0.001**
	Average health	967(51.8)	1106(56.3)	570(51.1)	2643(53.4)	
	Healthy	345(18.5)	438(22.3)	238(21.3)	1021(20.6)	
Health trend	Worse	168(9.0)	181(9.2)	102(9.1)	451(9.1)	**0.001**
	No change	832(44.6)	992(50.5)	558(50.0)	2382(48.1)	
	Better	867(46.4)	792(40.3)	456(40.9)	2115(42.8)	
Chronic disease	No	1196(64.1)	1421(72.3)	840(75.3)	3457(69.9)	**<0.001**
	Yes	671(35.9)	544(27.7)	276(24.7)	1491(30.1)	

**Table 2:** Association between Internet use and medical institution choices

Variables	Categories	Community health service centers (reference group: general hospitals)		Other medical institutions (reference group: general hospitals)	
		*P*	OR(95%CI)	*P*	OR(95%CI)
Internet use	Yes vs. No	**< 0.001**	0.667(0.558,0.797)	0.118	0.851(0.695,1.042)
Age (year)	65–69 vs. 60–64	0.212	0.897(0.757,1.064)	0.656	0.957(0.789,1.161)
	70–74 vs. 60–64	**0.038**	0.816(0.673,0.989)	0.077	0.817(0.653,1.022)
	≥ 75 vs. 60–64	**< 0.001**	0.633(0.510,0.785)	**< 0.001**	0.564(0.435,0.732)
Sex	Male vs. Female	**0.001**	1.259(1.093,1.451)	**0.03**	0.834(0.709,0.982)
Marital status	Non-partnered vs. Partnered	**0.034**	1.241(1.017,1.515)	**0.007**	1.358(1.086,1.699)
	Others vs. Partnered	0.864	0.963(0.624,1.485)	0.062	1.53(0.979,2.39)
Education	Primary and Middle School vs. Uneducated	**0.007**	0.810(0.694,0.945)	0.226	0.895(0.748,1.071)
	High school and above vs. Uneducated	**< 0.001**	0.625(0.497,0.787)	0.67	0.945(0.729,1.226)
Residential area	Urban vs. Rural	**< 0.001**	0.529(0.460,0.609)	**< 0.001**	0.595(0.505,0.7)
Medical insurance	Yes vs. No	0.16	0.837(0.652,1.073)	0.112	1.235(0.952,1.602)
Annual income (CNY)	Continuous variable	**0.021**	0.977(0.959,0.997)	**< 0.001**	0.952(0.932,0.973)
Satisfaction	Neutral vs. Dissatisfied	0.153	1.258(0.918,1.724)	**0.05**	1.43(0.999,2.045)
	Satisfied vs. Dissatisfied	0.631	1.068(0.817,1.396)	0.573	1.093(0.802,1.49)
Trust score	6–8 score vs. 0–5 score	**< 0.001**	1.367(1.160,1.610)	0.36	0.916(0.76,1.105)
	9–10 score vs. 0–5 score	**0.004**	1.295(1.085,1.545)	0.793	1.027(0.842,1.253)
Medical fee (CNY)	Continuous variable	**< 0.001**	0.936(0.918,0.954)	**< 0.001**	0.948(0.927,0.97)
Health status	Average healthy vs. Unhealthy	**< 0.001**	1.442(1.210,1.719)	0.815	0.977(0.8,1.191)
	Healthy vs. Unhealthy	0.106	1.205(0.961,1.51)	0.259	0.862(0.666,1.115)
Health trend	No change vs. Worse	0.369	1.116(0.879,1.417)	0.331	1.147(0.87,1.513)
	Better vs. Worse	0.753	0.961(0.751,1.23)	0.645	0.935(0.701,1.246)
Chronic disease	Yes vs. No	0.453	0.942(0.805,1.102)	**< 0.001**	0.707(0.587,0.851)

**Table 3:** Association of Internet use with medical institution choices grouped by confounding factors

Stratified variable		Community health service centers (reference group: general hospitals)		Other medical institutions (reference group: general hospitals)	
		*P*	OR(95%CI)	*P*	OR(95%CI)
Age (year)	60–64	0.082	0.777(0.585,1.032)	0.474	0.891(0.649,1.222)
	65–69	**< 0.001**	0.531(0.387,0.729)	0.4	0.859(0.602,1.224)
	≥ 70	0.075	0.735(0.517,1.032)	0.419	0.842(0.554,1.279)
Sex	Female	**< 0.001**	0.592(0.444,0.788)	0.135	0.794(0.587,1.074)
	Male	**0.004**	0.712(0.566,0.896)	0.518	0.912(0.691,1.204)
Marital status	Partnered	**< 0.001**	0.633(0.523,0.766)	**0.039**	0.794(0.637,0.989)
	Non-partnered	0.919	1.031(0.570,1.865)	0.354	1.361(0.709,2.611)
	Others	0.654	1.350(0.364,5.001)	0.189	2.524(0.635,10.038)
Education	Uneducated	0.141	0.727(0.476,1.111)	0.38	1.228(0.776,1.942)
	Primary and middle School	**< 0.001**	0.605(0.474,0.773)	**0.013**	0.699(0.528,0.926)
	High school and above	0.208	0.788(0.544,1.142)	0.99	0.997(0.662,1.504)
Residential area	Rural	0.331	0.862(0.639,1.163)	0.624	0.917(0.649,1.297)
	Urban	**< 0.001**	0.576(0.458,0.725)	0.185	0.839(0.647,1.088)
Medical insurance	No	**< 0.001**	0.654(0.545,0.786)	**0.045**	0.805(0.652,0.995)
	Yes	0.669	0.837(0.370,1.892)	0.364	1.438(0.656,3.151)
Satisfaction	Dissatisfied	**0.031**	0.437(0.205,0.929)	0.456	0.728(0.316,1.679)
	Neutral	**0.018**	0.543(0.328,0.900)	0.702	1.108(0.656,1.870)
	Satisfied	**0.001**	0.704(0.577,0.858)	0.071	0.808(0.641,1.019)
Trust score	0–5 score	**0.009**	0.635(0.451,0.893)	0.969	0.993(0.698,1.412)
	6–8 score	**0.005**	0.685(0.528,0.890)	0.003	0.613(0.443,0.849)
	9–10 score	**0.03**	0.663(0.458,0.961)	0.665	1.094(0.729,1.639)
Health status	Unhealthy	0.591	0.895(0.597,1.342)	0.654	0.908(0.594,1.386)
	Average healthy	**< 0.001**	0.621(0.491,0.786)	0.305	0.867(0.660,1.139)
	Healthy	**0.008**	0.591(0.400,0.874)	0.146	0.713(0.451,1.126)
Health trend	Worse	0.849	0.937(0.482,1.823)	0.419	1.354(0.649,2.823)
	No change	**< 0.001**	0.620(0.487,0.789)	0.032	0.737(0.558,0.974)
	Better	**0.009**	0.673(0.499,0.907)	0.857	0.970(0.696,1.351)
Chronic disease	No	**< 0.001**	0.646(0.521,0.801)	0.221	0.862(0.679,1.094)
	Yes	0.059	0.734(0.532,1.011)	0.405	0.845(0.57,1.255)

## Discussion

Based on 4,948 samples from the 2020 CFPS data, this study investigated the relationship between Internet use and the choice of medical institution among older adults aged ≥ 60 years. The use of the Internet enhanced the likelihood of older individuals accessing general hospitals rather than community health service centers. Furthermore, this association remained consistent across various factors, such as age of 65–69 years, having a partner, primary or secondary education level, residing in urban areas, absence of medical insurance, average or healthy self-rated health status, unchanged or better health trend, and absence of chronic disease. Overall, the research findings are relevant to the application and promotion of Internet use in medical services.

### Association between internet use and medical institution choice

Consistent with previous studies, the results showed that Internet use makes older people more likely to choose general hospitals rather than community health service centers for medical treatment for their daily medical needs. In China, Internet use increased the likelihood of residents of different age groups seeking care at more advanced healthcare facilities [16–18, 23]. Currently, patients of almost all ages use the Internet as their primary source of health information [24, 25]. However, health information available on the Internet is complex. On the one hand, older adults lack the ability to discriminate, and they become confused. However, information overload causes information anxiety and increases cognitive load. Older adults are more susceptible to health anxiety due to the misunderstanding of specialized medical knowledge or incomplete information [24]. In addition, to minimize health-related anxiety, people engage in activities they perceive as safe [25]. This also suggests that older people who are Internet users may be more inclined to contact advanced healthcare professionals.

Regarding the confounding factors, characteristics such as older age, higher education level, higher annual income, higher medical fees, and chronic disease were associated with a higher likelihood of seeking care in general hospitals. A Chinese study reported the association between age, education, and income and healthcare preferences, with higher earners who value technology being more likely to choose general hospitals [26]. However, older adults with lower income preferred community-based primary care facilities [27]. In addition, belonging to the male sex, being widowed, and having a good health status were all variables that encouraged older individuals to seek care at community health service centers. Furthermore, greater trust in doctors made them prefer community health service centers, consistent with the findings of Lu et al. [28]. Moreover, trust in doctors can alleviate patients’ uncertainty regarding healthcare information [29]. This highlights the enduring importance of doctor–patient trust in choosing medical institutions, even with widespread applications of the Internet and digital health systems.

Subgroup analyses were conducted by age, sex, marital status, education, residential area, medical insurance, satisfaction, trust score, health status, health trend, and chronic disease to reveal differences. In terms of age and residential area, users aged 60–69 years or those residing in urban areas were more prone to broadening their social networks and accessing diverse online information [30]. However, Internet use may diminish life satisfaction, resulting in negative attitudes [30]. In contrast, rural older people live in relatively closed social networks of acquaintances and lack reliable Internet access [31–33]. They select healthcare based on health status [29], insurance coverage [31], support systems [32], long-term care [33], and resource availability. In terms of education, less-educated older Internet users were more susceptible to Internet dependency upon exposure. Insufficient eHealth literacy may impede their medical decision-making [34]. In addition, older users with a better health status spent more time and effort seeking online health information. While researching their own or their loved ones’ medical issues, they may engage in negative rumination [35]. Health information received can lead to changes in behavior and healthcare choices. Regarding chronic diseases, Internet use increased the likelihood of older adults without chronic disease opting for general hospitals for their regular medical needs; however, no such impact observed among those with chronic illnesses. Those without chronic conditions have less frequent medical needs but may be more susceptible to illnesses. When exposed to health information on the Internet, they are easily misled or interfered with by various sources and unverified related information, which exaggerates the severity of their symptoms [36]. Chronically ill older patients require regular follow-up visits or treatment [37, 38]; this provides a clearer understanding of the service quality of the community health center. To some extent, this can counteract the Internet’s influence on the choice of medical institution. Owing to the decline in physical capabilities, older people have a higher demand for health information than younger individuals [21]. Information noise is prevalent in the barbaric development stage of Internet healthcare, with fewer reliable disease information websites than those promoting dubious health advice [39]. Inconsistent and incomplete online health information blurs the cognitive boundaries between minor and serious diseases [17]. Older adults’ interpretation and judgment of information can affect their choice of medical institution.

### Limitations

This study focused on older adults and offered insights into the association between the Internet use and the choice of medical institution in different subgroups. However, this study has certain limitations. First, this cross-sectional study could only identify correlations and could not establish causality. Second, most older adults are light Internet users with limited initiative, which leads to passive exposure to health information and makes it difficult to strictly distinguish health information-seeking from Internet use. Third, we adjusted for confounding factors and conducted a stratified analysis. The distribution is inconsistent among the variables analyzed, which may have a negative impact on the robustness of the results. Therefore, the results of this study may not apply to all situations. Fourth, due to the limitations of the questionnaire, we were unable to control for the effect of disease severity on medical institution choice and obtain more details about Internet use from the database.

### Conclusion

This study, conducted on a nationwide sample of older Chinese individuals, implied that Internet use significantly increased the probability of older individuals selecting a general hospital. The subgroup analyses indicated that this association differed among older adults with different characteristics. Future research could further explore the impact of Internet use on behaviors related to medical consultations and the utilization of online healthcare services.

## Data Availability

The data used in this study were obtained from a public survey released by the China Social Science Survey Center (ISSS) of Peking University and are available at http://www.isss.pku.edu.cn/cfps/index.htm.

## Abbreviations

CFPS: China Family Panel Survey
